# Effect of dosing interval on the pharmacokinetic interaction between montmorillonite powder and pyrotinib in rats

**DOI:** 10.3389/fphar.2026.1875749

**Published:** 2026-08-12

**Authors:** Xiaohuan Gao, Guangzhao He, Yanzhi Bi, Hui Luo, Jing Wu, Yuanyuan Zu, Yun Ji, Jingping Yu

**Affiliations:** 1 Central Operating Room, Changzhou Cancer Hospital, Changzhou, China; 2 Office of Clinical Trial Institution, Changzhou Cancer Hospital, Changzhou, China; 3 Department of Medical Oncology, Changzhou Cancer Hospital, Changzhou, China; 4 Department of Pharmacy, Changzhou Cancer Hospital, Changzhou, China; 5 Department of Radiotherapy, Changzhou Cancer Hospital, Changzhou, China

**Keywords:** drug-drug interaction, liquid chromatography-tandem mass spectrometry, montmorillonite powder, pharmacokinetics, pyrotinib, rat

## Abstract

**Aims:**

Montmorillonite powder (MP) can adsorb pyrotinib and reduce its bioavailability. This study investigated the effects of different dosing intervals on the pharmacokinetic interaction between MP and pyrotinib in rats.

**Methods:**

Eighteen male Sprague-Dawley rats were randomly assigned to three groups (n = 6/group): Control (pyrotinib alone), 0.5-h interval (MP given 0.5 h after pyrotinib), and 2.0-h interval (MP given 2.0 h after pyrotinib). Pyrotinib (10 mg/kg) was orally administered to all rats, followed by MP (240 mg/kg) or water (control) at designated time points. Blood samples were collected up to 24 h post-dose. Plasma pyrotinib concentrations were determined by a validated liquid chromatography-tandem mass spectrometry method. Pharmacokinetic parameters were calculated using WinNonlin.

**Results:**

The 0.5-h interval group showed substantially reduced pyrotinib exposure, with area under the curve (AUC) from time zero to last time point (AUC_last_) and AUC from time zero to infinity (AUC_inf_) decreasing by 31.2% and 31.3%, respectively, indicating statistically significant reductions (90% CIs <1.0). *C*_max_ reduction was 20.7%, which was not statistically significant (90% CI crossed 1.0). For the 2.0-h interval group, all GMRs were close to unity, but the wide 90% CIs (69.1%–137.6%) could not rule out a clinically meaningful impact.

**Conclusion:**

In rats, MP administration at an insufficient dosing interval markedly compromises pyrotinib systemic exposure, whereas adequate temporal separation largely mitigates this interaction. These findings underscore the urgent need for dedicated clinical pharmacokinetic studies to define the optimal dosing interval in humans and to minimize the risk of subtherapeutic pyrotinib exposure in clinical practice.

## 1 Introduction

Montmorillonite powder (MP) is a commonly used oral clay adsorbent that is clinically indicated for the treatment of diarrhea, gastrointestinal inflammation, and drug poisoning (Khediri et al., 2011). Its primary mechanism of action involves physical adsorption of gastrointestinal contents through its layered silicate structure via electrostatic interactions, hydrogen bonding and intercalation, thereby reducing exposure to harmful substances or excessive drugs (Damato et al., 2022). These mechanisms primarily favor cationic and basic drugs.

MP may also affect the absorption of co-administered drugs due to its adsorptive properties. *In vitro* studies have confirmed that MP can strongly adsorb drugs such as norfloxacin and ranitidine (Castela-Papin et al., 1999; Xiang, 2015). Pyrotinib an oral tyrosine kinase inhibitor for human epidermal growth factor receptor 2 positive recurrent or metastatic breast cancer (Xu et al., 2021; Zheng et al., 2023). Pyrotinib (pKa 8.71) is a weakly basic drug containing a pyridine ring and a quinoline ring, similar to norfloxacin, suggesting potential for adsorption by MP (Liu, 2022; Zelaya Soulé et al., 2021). However, pyrotinib has a larger molecular weight (583 Da) and a more complex aromatic structure, which may affect intercalation efficiency and adsorption kinetics. To date, no published *in vitro* study has specifically evaluated pyrotinib adsorption by MP, representing a knowledge gap in the literature.

Regarding its disposition, pyrotinib is an orally administered drug that is primarily metabolized by CYP3A4 (contributing >90%), and to a lesser extent by CYP1B1, CYP2C8, CYP2C19, CYP2D6, and CYP3A5 (Zhu et al., 2016). There is no definitive evidence that pyrotinib is a substrate of efflux transporters such as P-glycoprotein or breast cancer resistance protein. Although MP is unlikely to systemically inhibit these enzymes or transporters due to its negligible oral absorption and lack of tissue accumulation, its altered absorption kinetics could indirectly affect the pharmacokinetics of co-administered oral drugs, even when dosed at certain intervals (Wang et al., 2025; Wen et al., 2021).

Given that systemic exposure to oral tyrosine kinase inhibitors is critical for both efficacy and safety, pyrotinib has been proposed as a candidate for therapeutic drug monitoring (van der Kleij et al., 2025; Zhang et al., 2026; Gao et al., 2025). However, therapeutic drug monitoring alone cannot address potential pharmacokinetic interactions with co-administered drugs. MP is commonly used to manage pyrotinib-induced diarrhea (Wang et al., 2025; Wen et al., 2021; Fang et al., 2022). Previous clinical studies have indicated that even when MP is administered 2.0 h after a single dose of pyrotinib in healthy volunteers, the area under the curve (AUC) of pyrotinib is still reduced by 33.1% (AUClast) and 32.4% (AUC_inf_) (Wang et al., 2025). Population pharmacokinetic analyses have further shown that montmorillonite can decrease the bioavailability of pyrotinib by about 50% in breast cancer patients (Wen et al., 2021). Such significant reductions in AUC may compromise the antitumor efficacy of pyrotinib, warranting cautious use of MP during pyrotinib treatment (Wang et al., 2025; Wen et al., 2021). Nevertheless, MP remains an important agent for managing pyrotinib-induced diarrhea. Therefore, a critical clinical challenge is how to maximize the antidiarrheal benefit of MP while minimizing its negative impact on the absorption of pyrotinib (Wang et al., 2025; Wen et al., 2021; Fang et al., 2022).

The present study aims to investigate the effect of different dosing intervals on the pharmacokinetic interaction between MP and pyrotinib in rats, thereby providing a preclinical reference to inform the design of future clinical studies on the optimal timing of MP administration in the management of pyrotinib-induced diarrhea.

## 2 Methods

### 2.1 Chemicals and reagents

MP (batch No. W00999) was purchased from Beaufour Ipsen (Tianjin) Pharmaceutical Co., Ltd. (Tianjin, China). Pyrotinib (batch No. A302849, purity 99.89%) and buspirone (internal standard, IS, batch No. T580537, HPLC ≥99%) were obtained from Aladdin Biochemical Technology Co., Ltd. (Shanghai, China). The chemical structures of montmorillonite (PubChem SID: 404771185), pyrotinib (PubChem CID: 51039030), and buspirone (PubChem CID: 2477) were retrieved from the PubChem database and are depicted in Supplementary Figures S1–S3, respectively (Kim et al., 2025). Methanol (HPLC grade), acetonitrile (HPLC grade), and Tween-80 (batch No. 9004-32-4) were purchased from Sigma-Aldrich (St. Louis, MO, United States). Formic acid (HPLC grade) was obtained from DikmaPure (Shanghai, China). All other reagents were of analytical grade and commercially available. Purified water was prepared in the laboratory.

### 2.2 Animals

Male Sprague-Dawley (SD) rats (6–8 weeks old, weighing 250 ± 10 g) were used in this study. A total of 18 rats were purchased from Vital River Laboratory Animal Technology Co., Ltd. (Zhejiang, China; license No. SCXK (Zhejiang) 2024-0001). All animals were housed at the Experimental Animal Center of Bioduro Biotechnology (Wuxi, China; animal use license No. SYXK (Jiangsu) 2025-0054) under a 12-h light/dark cycle with free access to food and water. Before drug administration, rats were fasted for 12 h but allowed free access to water. This animal study was approved by the Animal Ethics Committee of Bioduro Biotechnology (approval No. BDW-2501-0007).

### 2.3 Instruments

The liquid chromatography-tandem mass spectrometry (LC-MS/MS) system consisted of an LC-30AD ultra-high-performance liquid chromatograph (Shimadzu, Kyoto, Japan) coupled with an AB Sciex Triple Quad 6500 mass spectrometer (SCIEX, Framingham, MA, United States). A Sorvall ST 40R refrigerated benchtop centrifuge (Thermo Fisher Scientific, Waltham, MA, United States), MCA-C electronic balance (Sartorius, Göttingen, Germany), Vortex-Genie 2 vortex mixer (Scientific Industries, New York, NY, United States), and an ultra-low temperature freezer (Thermo Fisher Scientific) were also used.

### 2.4 LC-MS/MS conditions

#### 2.4.1 Chromatographic conditions

Chromatographic separation was performed on a Kinetex C_18_ column (50 mm × 2.1 mm, 2.6 µm; Phenomenex, Torrance, CA, United States). Mobile phase A consisted of 0.1% formic acid in water, and mobile phase B consisted of 0.1% formic acid in acetonitrile, which was in accordance with the reference (Zhang et al., 2026). The flow rate was 0.6 mL/min, the column temperature was ambient, and the injection volume was 1 µL. The gradient elution program was as follows: 0–0.30 min, 2% B; 0.30–1.50 min, 2%–98% B; 1.50–2.20 min, 98% B; 2.20–2.21 min, 98%–2% B; and 2.21–3.00 min, 2% B. No significant co-elution of endogenous matrix components with pyrotinib or IS was observed. Ion suppression was evaluated by post-column infusion and was found to be negligible (matrix effect <8%).

#### 2.4.2 Mass spectrometric conditions

The main mass parameters were consistent with a previous study (Zhang et al., 2026; Chai et al., 2021). Mass spectrometry was performed using electrospray ionization in positive ion mode with multiple reaction monitoring. To obtain the most prominent precursor-to-product ion transitions and to achieve the required sensitivity of the analyte in the biological matrix, full mass spectrometric parameters were optimized as follows: ion spray voltage, 5500 V; declustering potential, 10 V; ionization temperature, 500 °C; nebulizer gas (gas 1), 50 kPa; auxiliary gas (gas 2) 50 kPa; curtain gas, 40 kPa; collision gas, 6 kPa. Unit mass resolution was maintained for both quadrupoles. The precursor-to-product ion transitions were m/z 583.3 → 138.2 for pyrotinib and m/z 386.2 → 122.2 for IS, which was in accordance with the reference (Zhang et al., 2026; Chai et al., 2021). Declustering potentials were 30 eV for pyrotinib and 91 eV for IS; collision energies were 34 eV and 49 eV, respectively. The dwell time was 100 m per transition.

Buspirone was selected as the IS due to its structural similarity to pyrotinib, which yielded similar chromatographic behavior and stable ionization under ESI positive mode.

### 2.5 Preparation of standard and quality control samples

Pyrotinib and IS were accurately weighed and dissolved in dimethyl sulfoxide, then diluted with isopropanol to obtain 1.00 mg/mL stock solutions. Pyrotinib working solutions were prepared by serial dilution of the stock solution with methanol to concentrations of 10, 20, 50, 100, 200, 1000, 5,000, and 10,000 ng/mL for calibration curves, as well as 10 ng/mL (lower limit of quantification, LLOQ), 20 ng/mL (low quality control, LQC), 2000 ng/mL (medium quality control, MQC), and 8000 ng/mL (high quality control, HQC). For plasma calibration, 45 µL of blank plasma was spiked with 5 µL of the above working solutions to obtain final concentrations ranging from 1 to 1000 ng/mL. Quality control plasma samples were similarly prepared at 1 ng/mL (LLOQ), 2 ng/mL (LQC), 200 ng/mL (MQC), and 800 ng/mL (HQC). The IS working solution (5 ng/mL) was prepared by diluting the stock solution with methanol–acetonitrile (1:1, v/v). For plasma sample preparation, 50 µL of plasma was aliquoted, and protein precipitation was performed by adding 200 µL of internal standard solution (5 ng/mL) in methanol/acetonitrile (1:1, v/v). Prior to precipitation, 5 µL of methanol was added to all samples. The mixture was vortexed for 1 min and then centrifuged at 4500 rpm for 15 min. The resulting supernatant was subsequently transferred for LC-MS/MS analysis.

### 2.6 Method validation

The method was validated according to the guidelines of the Chinese Pharmacopoeia (2020 edition). Validation parameters included selectivity, calibration curve, lower limit of quantification, accuracy, precision, matrix effect, extraction recovery, stability, and dilution reliability.

### 2.7 Animal dosing and blood sampling

Eighteen SD rats were randomly divided into three groups (n = 6 per group) and administered drugs by oral gavage. Pyrotinib was dissolved in 0.5% Tween-80 solution to obtain a 2 mg/mL oral solution. MP was suspended in purified water (one sachet in 50 mL) by vortex mixing. The three groups were treated as follows: (1) Control group: pyrotinib (10 mg/kg) followed by purified water; (2) 0.5-h interval group: pyrotinib (10 mg/kg) followed by MP (240 mg/kg) at 0.5 h after pyrotinib; (3) 2.0-h interval group: pyrotinib (10 mg/kg) followed by MP (240 mg/kg) at 2.0 h after pyrotinib. The 0.5-h interval in rats was selected to represent a clinically insufficient separation in humans (approximately equivalent to a 2-h interval in patients), whereas the 2.0-h interval in rats was designed to reflect a sufficiently long separation in humans, given the considerably faster gastric emptying in rats than in humans (Wang et al., 2025; Wang et al., 2024).

Blood samples (approximately 100 µL each) were collected from the jugular vein without anesthesia before (0 h) and at 0.25, 0.5, 1, 2, 4, 6, 8, and 24 h after pyrotinib administration. Samples were placed in tubes containing dipotassium ethylenediaminetetraacetic acid, kept on ice, and centrifuged at 6,000 rpm for 5 min at 4 °C within 30 min of collection to obtain plasma. Plasma samples were temporarily stored on dry ice and then transferred to an −80 °C freezer until analysis. Food was provided starting at 4 h post pyrotinib dose. All rats were euthanized by carbon dioxide asphyxiation after the final blood collection.

### 2.8 Pharmacokinetic and statistical analysis

Pharmacokinetic parameters, including time to maximum concentration (*t*_max_), maximum concentration (*C*_max_), elimination half-life (*t*_*1/2*_), AUC_last_, AUC_inf_ and mean residence time (MRT), were calculated using WinNonlin software (version 8.2; Certara, Princeton, NJ, USA). AUC extrapolation from the last measured time point to infinity was performed using the standard log-linear trapezoidal method, with extrapolation percentage less than 20% for all animals.

Statistical analyses were performed using SPSS version 24.0 (SPSS Inc., Chicago, IL, United States). Plasma concentration–time curves were plotted using GraphPad Prism version 7.0 (GraphPad Software, San Diego, CA, United States) and data are presented as mean ± standard deviation (SD). The Kruskal-Wallis H test was used for three-group concentration comparisons at each time point. Individual pharmacokinetic parameters of pyrotinib (AUC_last_, AUC_inf_ and *C*_max_) were calculated using non-compartmental analysis based on plasma concentration data collected at eight time points per rat, and summarized descriptively by group. To estimate the differences in least-squares means among the three treatment groups, a general linear model was fitted to the log-transformed pharmacokinetic parameters, with treatment group as a fixed effect. As each rat contributed a single parameter value derived from the eight sampling time points, no random effect was included in the model. The results were exponentiated to the original scale to obtain geometric least-squares means, geometric mean ratios (GMRs) relative to the pyrotinib-alone group (control), and their corresponding 90% confidence interval (CI).

## 3 Results

### 3.1 Method validation

**FIGURE 1:**
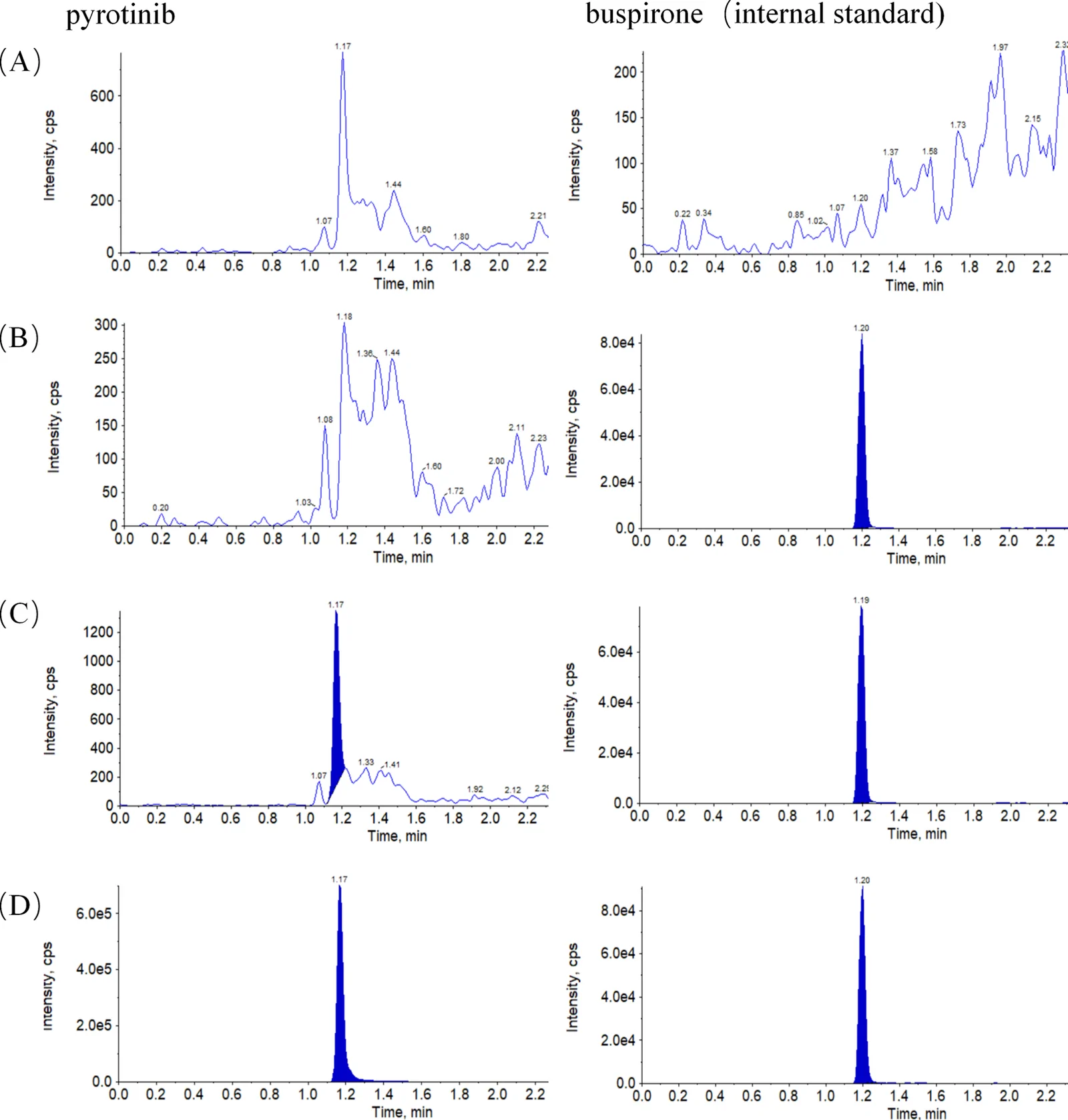
Representative chromatograms of pyrotinib and internal standard (buspirone) in rat plasma. **(A)** Blank rat plasma; **(B)** Blank plasma spiked with internal standard; **(C)** Plasma at the lower limit of quantification; **(D)** Plasma from the control group 2.0 h post-pyrotinib administration.

The retention times of pyrotinib and the IS were both approximately 1.2 min. The detection channels for the two analytes did not interfere with each other, and endogenous substances in the plasma did not affect the determination, indicating good specificity of the method. Figure 1 shows representative chromatograms of pyrotinib and the internal standard in different plasma samples.

Pyrotinib exhibited a good linear relationship in the concentration range of 1–1,000 ng/mL, with the regression equation: Y = 0.00952X − 0.00641 (*R* ^*2*^ = 0.9957).

The intra-day and inter-day precision values for the low-, medium-, and high-concentration quality control samples and LLOQ were all within 8.82%. The accuracies ranged from −5.86% to 7.98% for intra-day and from −5.56% to 6.41% for inter-day measurements.

The matrix effect factors for pyrotinib ranged from 95.71% to 106.35%, and the extraction recoveries ranged from 90.13% to 106.49%, indicating that the presence of the matrix did not interfere with the determination of pyrotinib.

Stability tests showed that under conditions of room temperature (25 °C) for 8 h, autosampler storage (15 °C) for 12 h, storage at −80 °C for 7 days, and three freeze–thaw cycles (−80 °C–25 °C), the relative errors were all less than 8.11% and the precision values were all less than 8.03%. These results indicate that the sample processing, storage, and measurement method have good stability.

For samples with concentrations exceeding the upper limit of the calibration curve, accurate determination could be achieved after dilution into the linear concentration range, with relative error values not exceeding ±8.31%, demonstrating that the dilution procedure did not affect the accuracy of the measurements.

### 3.2 Pharmacokinetic results

**FIGURE 2:**
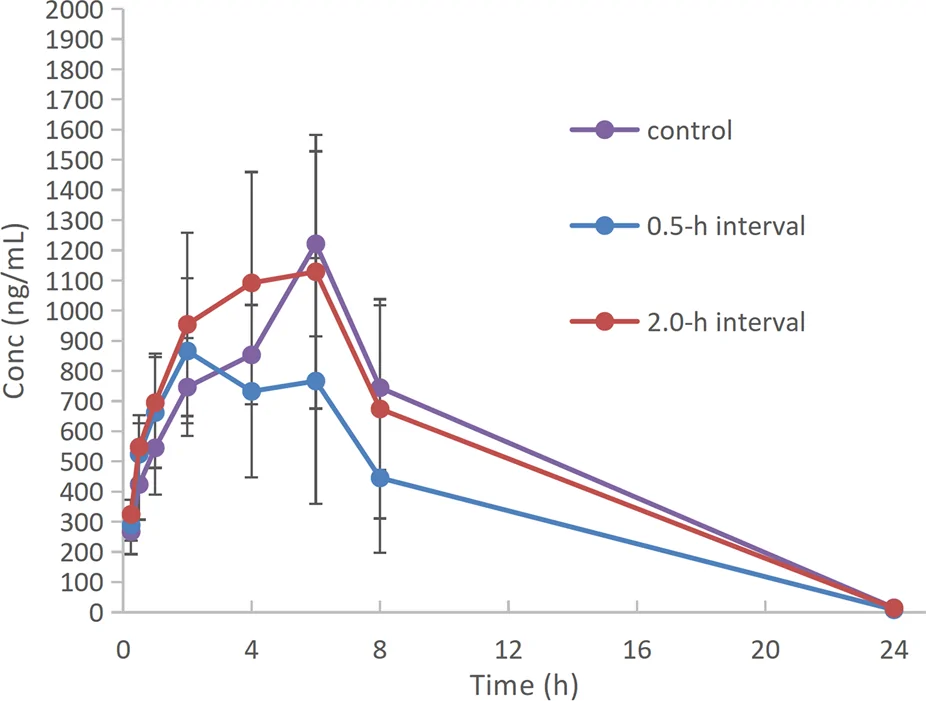
Plasma concentration-time curves (mean ± SD, n = 6) of pyrotinib in rats from the control group, 0.5-h interval group, and 2.0-h interval group.

The plasma concentrations of pyrotinib measured at each time point in the control group, the 0.5-h interval group, and the 2.0-h interval group are presented in Table 1. The concentration–time curves of pyrotinib for each group are shown in Figure 2, and the pharmacokinetic parameters of pyrotinib in the three groups are summarized in Table 2.

**TABLE 1:** Plasma concentrations of pyrotinib in rats at each time point following co-administration with montmorillonite powder at different dosing intervals (ng/mL, n = 6 per group).

Time points	Control	0.5-h Interval	2.0-h Interval
0.25	290 (257, 300)	297 (246, 335)	336 (315, 360)
0.50	415 (399, 466)	519 (474, 586)	534 (513, 568)
1.0	530 (443, 665)	644 (515, 790)	691 (593, 776)
2.0	738 (638, 823)	767 (718, 830)	897 (706, 1,088)
4.0	827 (748, 939)	649 (570, 730)	960 (826, 1,270)
6.0	1,285 (885, 1,475)	814 (564, 1,025)	986 (825, 1,178)
8.0	734 (612, 840)	430 (287, 601)	582 (410, 755)
24	9.2 (6.3, 14.4)	8.5 (5.4, 10.1)	15.1 (9.3, 21.2)

Data are presented as median (interquartile range).

**TABLE 2:** Pharmacokinetic parameters of montmorillonite powder combined with pyrotinib in rats (n = 6 per group).

Parameters	Control	0.5-h Interval	2.0-h Interval
AUC_last_(h·ng/mL)	12,368 (27)	8,513 (45)	12,074 (39)
AUC_inf_ (h·ng/mL)	12,436 (27)	8,543 (45)	12,128 (39)
*C*_max_(ng/mL)	1,184 (27)	842 (23)	1,170 (32)
*t*_max_ (h)^a^	6 (6, 6)	3 (2, 6)	5 (2, 6)
*t*_1/2_ (h)^b^	2.75 (0.69)	2.77 (0.10)	2.80 (0.35)
MRT_inf_ (h)	6.40 (0.47)	5.56 (0.79)	5.98 (0.42)

Data are geometric mean (geometric coefficient of variation).

^a^ For *t*_max_, median (minimum, maximum).

^b^ For *t*_1/2_, mean (SD). *t*_*1/2*_: Half-life of elimination, *t*_max_: Time to peak concentration, *C*_max_: Peak concentration, AUC_last_: Area under the curve from time zero to last time point, AUC_Inf_: Area under the curve from time zero to infinity, MRT_inf_: Mean residence time from time zero to infinity.

The blood sampling schedule (0–24 h) adequately captured the absorption, distribution, and terminal elimination phases, as evidenced by the return of plasma concentrations to near-baseline levels by 24 h in all groups. All plasma samples were above the LLOQ (1 ng/mL).

The Kruskal–Wallis H test of the pooled concentration data across all time points revealed no statistically significant difference in pyrotinib plasma concentrations among the three groups (H = 2.3913, df = 2, p = 0.3025). This result indicates that MP co-administration, irrespective of whether given at a 0.5-h or 2.0-h interval, did not produce a statistically significant overall alteration in the systemic exposure of pyrotinib in rats. Nevertheless, descriptive examination of the concentration–time profiles revealed certain numerical trends. At 4.0 h and 6.0 h post-dose, the 0.5-h interval group showed lower median concentrations than the control group, while the 2.0-h interval group exhibited higher median values at the corresponding time points.

As shown in Table 3, co-administration of MP at a 0.5-h interval resulted in GMRs of 68.8% (90% CI: 47.5%–99.7%) for AUC_last_, 68.7% (90% CI: 47.4%–99.6%) for AUC_inf_, and 79.3% (90% CI: 61.1%–103.0%) for *C*_max_, corresponding to reductions of 31.2%, 31.3%, and 20.7%, respectively, relative to pyrotinib alone. For the 2.0-h interval group, the GMRs were 97.6% (90% CI: 69.3%–137.6%) for AUC_last_, 97.5% (90% CI: 69.1%–137.5%) for AUC_inf_, and 98.6% (90% CI: 72.6%–133.9%) for *C*_max_, corresponding to minimal reductions of 2.4%, 2.5%, and 1.4%, respectively. Notably, he 0.5-h interval group showed significant AUC reductions (90% CI upper bounds <1.0); the 2.0-h group showed no significant differences (90% CIs crossed unity).

**TABLE 3:** Statistical comparison of pyrotinib plasma pharmacokinetic parameters for pyrotinib in different dosing intervals vs. control (pyrotinib alone).

Parameters	Geometric least-square mean			% ratio of least-square means(90% CI)	
	Control	0.5-h Interval	2.0-h Interval	0.5-h Interval vs. control	2.0-h Interval vs. control
AUC_last_(h·ng/mL)	12,300	8,510	12,100	68.8 (47.5, 99.7)	97.6 (69.3, 137.6)
AUC_inf_ (h·ng/mL)	12,400	8,540	12,100	68.7 (47.4, 99.6)	97.5 (69.1, 137.5)
*C*_max_(ng/mL)	1,180	842	1,170	79.3 (61.1, 103.0)	98.6 (72.6, 133.9)

Values were reported with three significant figures.

## 4 Discussion

This study provides the first experimental evidence, to our knowledge, of the time-dependent pharmacokinetic interaction between MP and pyrotinib in a controlled animal model. Unlike previous clinical studies that reported a 32.4% reduction in pyrotinib AUC without systematically evaluating different dosing intervals (Wang et al., 2025), the present study was specifically designed with two distinct MP dosing intervals (0.5 h and 2.0 h) to characterize the temporal relationship between MP administration and pyrotinib absorption.

The concentration-time profiles revealed numerical trends suggesting that a 0.5-h interval between MP and pyrotinib administration might be associated with reduced pyrotinib absorption during the peak concentration window (4.0–6.0 h), whereas a 2.0-h interval appeared to allow for relatively higher systemic exposure. These observations are mechanistically consistent with the adsorptive properties of MP, which may temporarily adsorb pyrotinib in the gastrointestinal tract when the two agents are administered in close temporal proximity; a longer interval may permit sufficient gastric emptying and intestinal dissolution before MP exerts its adsorptive effect. However, these numerical differences did not reach statistical significance under the Kruskal–Wallis H test (p = 0.3025), likely attributable to the limited sample size (n = 6 per group) and substantial inter-individual variability as reflected in the wide interquartile ranges.

In our study, co-administration of MP at the 0.5-h interval reduced pyrotinib exposure, with AUC_last_ and AUC_inf_ decreasing by approximately 31%. Although the upper bounds of the 90% CIs were close to unity, the reductions reached statistical significance, suggesting that MP interferes with the oral absorption or systemic availability of pyrotinib. Given the established exposure-response relationship for pyrotinib, a 31% reduction in exposure may compromise therapeutic efficacy. The *C*_max_ reduction of 20.7% did not reach statistical significance, implying that the absorption rate might be less affected than the overall extent of absorption.

The 1.5-h prolongation of the dosing interval (from 0.5 to 2.0 h) substantially attenuated the interaction in the rat model, with all GMRs close to unity. Although the 90% CIs (ranged from 69.1% to 137.6%) were wide and failed to meet the strict 80%–125% equivalence criterion, the point estimates of GMRs were close to unity, suggesting that the extended dosing interval may allow near-complete pyrotinib absorption prior to MP reaching the gut. However, the wide CIs indicate that this interpretation remains uncertain and should be confirmed in future studies.

Notably, compared with the control group, *t*_max_ was shortened in the 0.5-h interval group and 2.0-h interval group. One possible explanation is that a larger proportion of pyrotinib had already been absorbed before MP administration in the interval groups compared with the control group, causing the absorption peak to occur earlier. This is consistent with our previous study of osimertinib, another oral tyrosine kinase inhibitor (He et al., 2026).

Regarding the mechanistic basis, pyrotinib is primarily metabolized by CYP3A4 (>90%) and is not a known substrate of efflux transporters. While MP is unlikely to systemically inhibit these enzymes or transporters due to its negligible oral absorption and lack of tissue accumulation, the observed reduction in AUC with a 0.5-h interval is more consistent with local gastrointestinal adsorption rather than systemic metabolic inhibition. The comparable *t*_max_ across groups further supports this interpretation, as an effect on systemic clearance would typically alter *t*_*1/2*_. Future *in vitro* adsorption studies under simulated gastrointestinal conditions are needed to directly quantify pyrotinib binding to MP and to elucidate the molecular mechanisms of intercalation and electrostatic interaction.

Although previous clinical studies have indicated that MP can reduce the bioavailability of pyrotinib, and such a marked reduction might compromise antitumor efficacy, necessitating cautious use of MP during pyrotinib treatment (Wang et al., 2025; Wen et al., 2021), these clinical studies did not consider whether the dosing interval between MP and pyrotinib was sufficient. Based on the results of the present study in rats, a longer dosing interval between MP and pyrotinib (e.g., 4 h) would likely exert minimal or negligible effect on pyrotinib absorption. Therefore, further pharmacokinetic interaction studies between MP and pyrotinib in humans are warranted to ensure that MP can be effectively used to manage pyrotinib-induced diarrhea without compromising pyrotinib bioavailability.

It is important to note that the present findings were obtained in a rat model. A previous clinical study reported a 32.4% reduction in pyrotinib AUC even with a 2.0-h interval (Wang et al., 2025), whereas in the present rat study, the 2.0-h interval largely prevented significant changes in AUC. This discrepancy may be explained by species differences in gastrointestinal transit time, gastric emptying rate, intestinal pH, and absorption kinetics. Therefore, the present findings should not be interpreted as clinical evidence that a 2.0-h interval is sufficient in humans; rather, they support the need for further clinical pharmacokinetic interaction studies with staggered administration.

Several limitations should be acknowledged regarding the translational relevance of the present findings. First, significant species differences exist in gastrointestinal physiology (e.g., transit time, gastric pH, motility) and in CYP enzyme expression (e.g., rat CYP3A1 vs. human CYP3A4), which could affect the presystemic metabolism of pyrotinib. The adsorption behavior of MP may also differ between species due to variations in gastrointestinal pH and luminal contents. These differences may substantially affect the absorption kinetics of both pyrotinib and MP, as well as their interaction profile. Therefore, the quantitative findings of this study, including the optimal 2.0-h interval, should not be directly extrapolated to humans without clinical validation. Second, the sample size of six animals per group is relatively small, and substantial interindividual variability was observed in some pharmacokinetic parameters, particularly AUC values. Consequently, the study may be underpowered to detect small differences. Third, the dose-linearity of pyrotinib pharmacokinetics in rats was not evaluated across different dose levels in this study. Nevertheless, the literature supports linear pharmacokinetic characteristics for pyrotinib (Ma et al., 2017; Li et al., 2017; Wang et al., 2022), suggesting that the possible effect of dose on the observed interaction magnitude, if any, may warrant caution in data interpretation. Fourth, only male rats were used to avoid potential confounding effects of the estrous cycle on drug absorption and disposition, and although no sex-related differences in pyrotinib pharmacokinetics have been reported to date (Li et al., 2017; Wang et al., 2022), hormonal influences on drug-metabolizing enzymes and gastrointestinal motility cannot be entirely excluded; consequently, the exclusion of female animals limits the generalizability of the findings to female patients, who constitute the majority of the HER2-positive breast cancer population. Additionally, the simultaneous administration of MP and pyrotinib was not evaluated, nor were pharmacodynamic endpoints assessed. Finally, given that circadian rhythms significantly influence drug absorption and disposition, the dosing time in this study (9:30 a.m., 2 h after lights on) fell during the rat’s rest phase, which may have contributed to variability in the observed pharmacokinetic parameters (He et al., 2026).

Future studies should validate the proposed mechanism using *in vitro* adsorption assays under simulated gastrointestinal tract conditions. The potential for biopharmaceutic interaction between MP and orally administered drugs, particularly those with adsorption-prone molecular structures, remains to be systematically characterized using controlled *in vitro* models (Weiss-Tessbach et al., 2025). Furthermore, therapeutic drug monitoring using a validated LC-MS/MS method could directly assess whether adsorbent administration alters pyrotinib plasma concentrations in patients (Zhang et al., 2026; Gao et al., 2025). Other oral clay adsorbents clinically indicated for diarrhea, gastrointestinal inflammation, and drug poisoning include diosmectite, kaolin, and attapulgite (Damato et al., 2022). These agents similarly exhibit adsorptive properties and have been reported to interact with various orally administered drugs, including tyrosine kinase inhibitors (He et al., 2026). The present findings may have broader implications for this class of adsorbents, highlighting the need for careful dosing interval optimization when these agents are used concomitantly with oral targeted therapies.

## 5 Conclusion

In conclusion, MP exerts a time-dependent inhibitory effect on the oral absorption of pyrotinib in rats. A 2.0-h interval between administrations largely mitigates this interaction in the rat model. This study provides a framework for designing future clinical pharmacokinetic interaction studies to determine the optimal dosing interval for the interaction between MP and pyrotinib to manage pyrotinib-induced diarrhea in humans. Further *in vitro* and clinical studies are warranted to confirm the pharmacokinetic impact of MP on pyrotinib.

## Data Availability

The original contributions presented in the study are included in the article/Supplementary Material, further inquiries can be directed to the corresponding authors.

## Supplementary material

SUPPLEMENTARY FIGURE S1 Chemical structure of montmorillonite (PubChem SID: 404771185), shown as a schematic representation of its 2:1 layered structure with interlayer cations.

SUPPLEMENTARY FIGURE S2 Chemical structure of pyrotinib (PubChem CID: 51039030), molecular formula C32H31ClN6O3, MW 583 g/mol.

SUPPLEMENTARY FIGURE S3 Chemical structure of buspirone (PubChem CID: 2477), molecular formula C21H31N5O2, MW 385.5 g/mol. All structures were retrieved from the PubChem database (Kim et al., 2025) and are reproduced for illustrative purposes.
